# Effects and safety of acne vulgaris with external application of herbal medicines: A protocol for systematic review and meta analysis

**DOI:** 10.1097/MD.0000000000026408

**Published:** 2021-07-02

**Authors:** Jin Zhou, Xiaoxiao Li, Haimin Chen, Zirong Qi, Shujun Shao, Yinshan Tang, Chenglong Jiang

**Affiliations:** a Hubei 672 Orthopaedics Hospital of Integrated Chinese & Western Medicine, Wuhan, 430079 Hubei; b Department of Nursing, The First People's Hospital of Foshan; c Guangdong Province Hospital of Integrated Traditional Chinese and Western Medicine, Foshan, Guangdong; d School of Acupuncture-Moxibustion and Tuina, Beijing University of Chinese Medicine, Beijing; e Department of Rehabilitation in Traditional Chinese Medicine, The Second Affiliated Hospital of Zhejiang University School of Medicine, Hangzhou, China.

**Keywords:** acne vulgaris, external application of herbal medicines, protocol, review, systematic

## Abstract

**Background:**

Acne vulgaris (AV) is a common dermatologic disease. The morbidity is increasing annually. External application of herbal medicines (EAHM) has been pervasively used in the therapy of AV. EAHM , as the traditional Chinese therapy, is widely applied in clinical trials for AV. The aim of this review is to systematically evaluate the efficacy and safety of EAHM in the therapy for AV.

**Methods:**

We will conduct an electronic search of 13 databases from their inception to May, 2020, including PubMed, EMBASE, MEDLINE, Web of Science, Cochrane Library, SpringerLink, WHO International Clinical Trials Registry Platform, Wanfang China database, China National Knowledge Infrastructure, Chinese Biomedical Literature Database, Chinese Scientific Journal Database, as well as China's Conference Papers Database and China Dissertation database. Other valid search strategies will also be retrieved to complete this review. All randomized controlled trials in which EAHM was used for the treatment of AV will be adopted. Two researchers will select eligible studies respectively according to a predefined protocol. Methodological quality will be assessed with Cochrane risk of bias by means of RevMan V.5.3.5 software.

**Results:**

This systematic view will present a high-quality synthesis based on current evidence of EAHM intervention for AV patients.

**Conclusion:**

The summary of our systematic view will provide evidence to judge whether EAHM is an effective and safe intervention for AV patients.

## 1 Introduction

Acne vulgaris (AV) is a chronic disease of the pilosebaceous follicles include sebaceous glands and hair follicles, usually occurring on the face, chest, back, as well as neck, inducing polymorph cutaneous lesions.^[1,2]^ The clinical features of acne, which is characterized by seborrhea, noninflammatory lesion such as closed and open comedones, inflammatory lesion such as pustule and papule, which may leave scars after regression.^[3]^ The clinical features of acne alternate with periods of exacerbation and stability.^[4]^

AV is a very common dermatologic disease, which has affected the majority of adolescents even accompanying them over 10 years. It ranks fourth in the list of patients aged 11–21 years old visiting a doctor in the United States.^[5,6]^ According to statistics, AV has affected 9.38% of the global population, making it the eighth-most prevalent disease worldwide, with the morbidity and the proportion of severity are increasing annually.^[7]^ Many papers have demonstrated that the incidence of acne is divided as to different countries and ethnic groups,^[8]^ for instance, in Germany the morbidity of AV is 26.8%,^[9]^ whereas the prevalence of acne skyrocket to 96% in Brazil.^[10]^ Depending on the factors, including region, subjects’ ages, and nationality, etc, the estimated incidence of acne in China ranges from 8.1% to 85.1%.^[11–13]^ According to a comprehensive systematic review, the morbidity of acne was 39.2% in Mainland China.^[14]^ Multiple studies indicate that there exists a strong association between acne presentation or severity and several risk factors, including demographic genetic factors, age, body mass index, and skin type. Whereas, there is a less clear correlation between acne presentation or severity and other factors, such as smoking and diet habits.^[15]^

Epidemiological studies have manifested that AV is the most common disease in postpubescent teens, especially boys have more severe forms.^[7]^ Individuals with AV have significant physical and psychological morbidity, lowering life quality, although it is not life-threatening. In terms of the potential harm of acne, it is acknowledged that there are 2 aspects: one contains physical impacts, such as symptomatic discomfort, scarring, and poor body image ^[16]^ and the other involves psychological impacts, such as low satisfaction with appearance, loss of self-confidence and self-esteem, and social isolation. Acne also can give rise to anxiety depression and suicide.^[17]^ Based on a high-quality meta-analytic review including 42 studies, we found AV was significantly tied up with depression and anxiety, indicating that individuals with acne have a high probability of suffering from anxiety and depression than those healthy individuals.^[17]^ Dermatologists should spare no efforts to pursue profound therapy of AV and consult psychologys^’^ referrals.

In America, the expenditure on treatment of AV approximates 3 billion dollars annually, which has induced a heavy fiscal burden on the health system as well as society.^[18]^ If we can seek out efficient approaches to cure AV, it will significantly relieve social burden as well as alleviate the adverse impacts on the patients, boosting their self-confidence and the life quality.

The pathogenesis of AV is multifactorial and the current understanding of it is continuously evolving. To summarize, the occurrence and progress of AV involve the following 5 main aspects: (1) massive secretion of sebum by the sebaceous gland; (2) other androgen-mediated activity; (3) follicular plugging due to abnormal keratinization of hair follicles and sebaceous glands; (4) bacterial proliferation of propionibacterium acnes (P acnes) within the follicle; and (5) inflammatory reaction and immunity;^[19,20]^ P acnes are thought to play a significant role in inflammation through induction of proinflammatory cytokines and recruitment of inflammatory cells.^[21]^ Besides, researches have indicated that the development of AV also attributes to neuroendocrine regulatory mechanisms, diet, nongenetic, and genetic factors.^[22]^

AV grading systems do good to specific grading, ensuring the most appropriate therapy plan and monitoring the development of patients’ condition during treatment. Recommendations for grading and classifying of AV are as follows: the first-line treatments for mild patients are benzoyl peroxide (BP) or topical retinoid or topical combination therapies, including BP add antibiotic or retinoid, or the combination of retinoid, BP and antibiotic; the first-line treatments for moderate conditions are topical combination therapy, including BP add antibiotic or retinoid or retinoid add BP add antibiotic or oral antibiotic add topical retinoid add BP or oral antibiotic add topical retinoid add BP add topical antibiotic; the first-line treatments for severe patients are oral antibiotic add topical combination therapy: BP add antibiotic or retinoid add BP or retinoid add BP add antibiotics or oral isotretinoin.^[22]^

In addition, physical therapies, such as glycolic acid peels, pulsed dye laser, salicylic acid peels, intralesional corticosteroid injections have positive effects on acne nodules. Complementary and alternative therapies, such as tea tree oil, herbal, and diet have a profound impact on the treatment of acne.^[23]^ Whereas, after a long-term medication. the clinical efficacy will decrease due to drug resistance or side effects. For example, the most common adverse effect of oral tetracycline is gastrointestinal distress, including nausea and esophagitis.^[24]^ Skin reaction ranks second in the list of side effects of tetracycline including rash, pruritus, and photosensitivity, the occurrence ranged from 0.42% to 30.5%. Tetracyclines also can cause tinnitus and dizziness. With long-term consumption and higher doses, tetracyclines will trigger off pigmentation of the mucous membranes, skin, and teeth, even autoimmune diseases and hypersensitivity reactions.^[23]^ The most common adverse effects of topical retinoids include peeling, erythema, irritation, dry skin, stinging, and burning.^[24,25]^ After long-term systemic administration of isotretinoin, the rate of colonization with *Staphylococcus aureus* will skyrocket, giving rise to minor skin infections, or lip or perioral abscesses, which is a serious complication on rare occasions.^[26,27]^ It is well known that isotretinoin invites teratogenic effects and retinoic acid embryopathy.^[23]^

In view of these mentioned adverse effects of AV, it is necessary to augment current therapy for mild-severe acne, and alternative treatment is an excellent choice. Herbal medicines, a characteristic therapy in Chinese and Korean, based on unique theories, have been applied internally and externally to cure various diseases over thousands of years. Meanwhile, physicians have accumulated abundant experiences and knowledge in the fight against skin disease.^[28]^ External treatment of herbal medicines includes applying drugs on the skin, exhibiting the strength of convenience, safety, comfort, and low cost than conventional medicines (CM).^[29]^

External application of herbal medicines (EAHM) have been widely applied in the treatment of AV. The published systematic reviews have demonstrated the benefits of EAHM for AV in clinical studies.^[28]^ However, only a small amount of studies were included in the systematic review and meta analysis, published by Korean Medicine, and the sample size is insufficient evidence to assess the efficacy and safety of EAHM with AV. We found the former meta analysis omits many randomized controlled trials (RCTs) in China databases and some new high quality well-designed. Multi-center RCTs for AV carried out in the recent 2 years can be included in this review. Therefore, it is fundamental to conduct a complete article to evaluate the effects and safety of AV with EAHM.

The principal objective of our review is to further systematically appraise the effects and safety of EAHM in AV treatment.

## 2 Methods

The registration number of the protocol report is CRD42020193830. The protocol is developed following the recommendations of the Preferred Reporting Items for Systematic Reviews and Meta-Analyses protocols (PRISMA-P) statement guidelines,^[29]^ we will strictly comply with it.

### 2.1 Eligible criteria for study selection

#### 2.1.1 Types of studies

We will assess the literature in accordance with the following items: participants, interventions, comparisons, outcomes. The RCTs of AV with EAHM, without additional treatment, will be included in this review. There is no restriction on language or publication status. Studies that evaluate or compare the effects among different components of EAHM will be excluded. Studies that lack a detailed description of the random process or method will be ruled out. Other types of studies, such as animal mechanism researches, reviews as well as theory studies, qualitative studies, and case reports will not be included.

#### 2.1.2 Types of participants

Participants diagnosed with AV, with no restrictions of age, gender, race, nationality, and educational background will be included.

#### 2.1.3 Types of interventions

##### 2.1.3.1 Experimental interventions

All types of EAHM will be included without the restriction of dosage, components, and duration of treatment, which are applied to the skin lesions. The herbal medicines included in the meta analysis are defined as any types of products that stemmed from botany, such as whole plants or their adjuncts.^[30]^ Studies that have combined effects of EAHM plus other interventions (eg, EAHM plus oral medicine, or acupuncture, etc) will be excluded.

##### 2.1.3.2 Control interventions

Clinical trials with no treatment, placebo, or CM will be considered as control groups. CM, for instance, zinc sulfate solution and benzoyl peroxide gel.^[28]^ Additionally, trials comparing EAHM with any other therapies will be excluded. What's more, the control interventions using unproven drugs such as herbal medicine will be ruled out.

#### 2.1.4 Types of outcome measures

##### 2.1.4.1 Primary outcomes

The primary outcome is the mean percentage reduction in the inflammatory lesion count (MPRILC), MPRILC = lesion number prior to treatment − lesion number following treatment)/lesion number prior to treatment × 100%).^[31,32]^ The inflammatory lesion count should be assessed by a dermatologist.

##### 2.1.4.2 Secondary outcomes

The secondary outcomes in the meta analysis are as follows:

1. The sebum and moisture of the skin.
2. Patient-reported changes in symptoms.
3. Quality of life.
4. Anti-bacterial activity test.
5. Adverse events, such as itching, irritation, erythema, and desquamation.

### 2.2 Search methods for the identifification of studies

#### 2.2.1 Electronic searches

A search will be done to identify literature from electronic databases, including PubMed, EMBASE, MEDLINE, Web of Science, Cochrane Library, SpringerLink, WHO International Clinical Trials Registry Platform (ICTRP), Wanfang China database, China National Knowledge Infrastructure, Chinese Biomedical Literature Database, Chinese Scientific Journal Database, as well as China's Conference Papers Database and China Dissertation database from inception to May, 2020 without language and publication limitations. The following search items will be adopted: acne vulgaris, acne, AV and external application, external treatment, external use, topical application, topical use, topical treatment, dermal, skin, gel, ointment, cream, spray, oil, cosmetic product and herbal, drug therapy, herbal treatment, and traditional treatment. The above-mentioned search keyword or combination items will be used. The equivalent search terms will be used in the Chinese database. The detailed search strategy is summarized in Table 1, and we will make appropriate adjustments for other databases.

**Table 1:** Search strategy for PubMed.

Number	Search terms
1	Acne
2	acne vulgaris
3	AV
4	1 or 2–3
5	external treatment
6	external use
7	external application
8	topical application
9	topical use
10	topical treatment
12	ointment
13	gel
14	spray
15	oil
16	Cosmetic product
17	skin
18	cream
19	dermal
20	5 or 6–19
21	herbal treatment
22	herbal
23	traditional treatment
24	plant extra
25	ethnobotany
26	traditional Chinese medicine
27	kampo medicine
28	traditional Korean medicine
29	21 or 22–28
30	Randomized controlled trial
31	Controlled clinical trial
32	randomized
33	randomly
34	placebo
35	Trial
36	30 or 31–35
37	4 and 20 and 29 and36

Besides, we will contact authors for the full article if the article is incomplete.

#### 2.2.2 Searching other resources

We will retrieve informal publications, the clinical trial registries, dissertations, and gray literature as a complement. Hand searches for the reference lists of identified relevant RCTs and reviews will be done to retrieve literature, ensuring all relevant literature are included in the review. We will also conduct a search to pick out ongoing or unpublished trials pertaining to the theme from the following clinical trial registries: the ICTRP, the National Institute of Health clinical registry ClinicalTrials.gov, the Chinese clinical registry, and the Australian New Zealand Clinical Trials Registry. We will conduct hand search for potential gray articles such as unpublished conference literature.

### 2.3 Data collection and analysis

#### 2.3.1 Study selection

**Figure 1:**
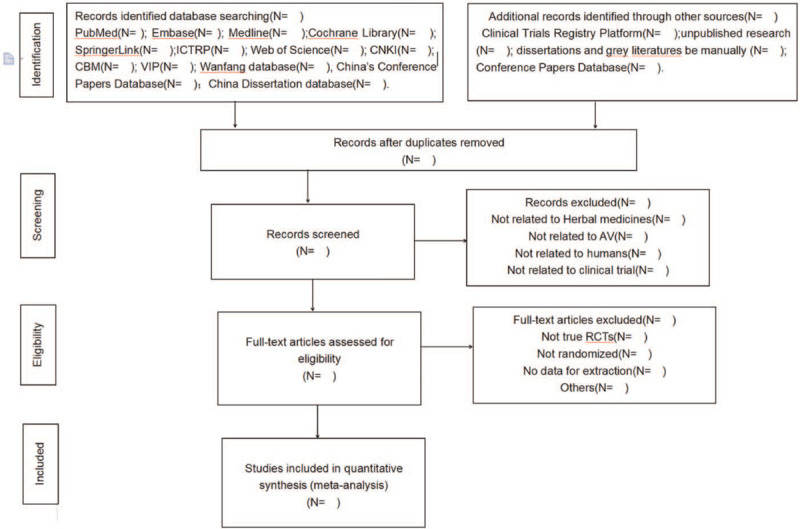
Preferred Reporting Items for Systematic Reviews and Meta-Analysis flow diagram.

Before the conduction of this study, all reviewers are trained by a qualified teacher and assure reviewers comprehensively understand the background and purpose of the review. Two authors (JZ and XL) will respectively screen the titles, abstracts, and keywords for eligible literature and remove duplications. By reviewing the full-text articles and analysis considerations, we can identify eligible studies. All reviewers need to examine the authors’ name, journal of publication, as well as their institutions. All studies that reviewers chose with final inclusion of studies to be decided through consensus, and judged by a third reviewer (YT) when discrepancies cannot be resolved through group discussion. The primary selection process is fully elucidated in a PRISMA flow chart (Fig. 1).

#### 2.3.2 Data extraction and management

According to predefined criteria, 2 authors (JZ and XL) will extract data independently. In every study, the following data will be extracted: publication year, authors name, design, sample size, methodological description, description of interventions, inclusion and exclusion criteria, characteristics of participants, follow-up duration, funding, outcomes, adverse effects, and other specific information, taking advantage of extraction form. All data will be cross-checked by JZ and XL, then reviewers will transfer them into Review Manager Software (RevMan V.5.2.1). Any disagreements will be settled through discussion, in addition, we can consult the senior reviewer (YT) to deal with these discrepancies and uncertainties. When the reported data are insufficient, we will contact authors for providing original data.

#### 2.3.3 Assessment of bias risk and quality of included studies

Two independent authors (HC and ZQ) will weigh the risk of bias assessment for eligible studies, based on the guidelines of the Cochrane Handbook for Systematic Reviews of Interventions.^[33]^ Risk of bias assessment categories will include the following: random sequence generation, allocation concealment, blinding of outcome assessment and participants, incomplete outcome data, and selective outcome reporting as well as other potential sources of bias. The assessments for each item will be graded as low bias, unclear, or high bias. The arbiter (YT) will do the final judge for inconsistent results. Clinical trial accords with all criteria will be graded as a low bias; a trial will be assessed as a high bias which conforms to none of the criteria; without sufficient information, we will contact the author for complete data, if it fails, the trail will be evaluated as unclear bias.

#### 2.3.4 Measurements of treatment effect

Continuous data will be presented as the mean difference to analyze the treatment effect, for the dichotomous outcomes, we will utilize relative risk. Both of them will be evaluated with a 95% confidence interval to indicate the effect size.

#### 2.3.5 Managing missing data

If data information are detected missing or incomplete, the 2 reviewers (SS and MX) will contact relevant authors for requesting missing data or insufficient data. If we fail to receive the necessary information, the data will be excluded from our analysis. At the same time, a group meeting will be organized to discuss the potential impact of the missing data and be addressed in the discussion section.

#### 2.3.6 Assessment of heterogeneity

Statistical heterogeneity across the studies included will be evaluated using χ^2^ test and *I*^2^ statistic. If the *P* < .1 of the χ^2^ test or *I*^2^ > 50%, indicating there exist substantial heterogeneity, and a random-effect model will be selected. Otherwise, a fixed-effect model will be employed.

#### 2.3.7 Assessment of reporting biases

If more than 10 RCTs are included in a meta-analysis, a visual asymmetry on the funnel plots will be employed to evaluate the potential reporting biases.

#### 2.3.8 Pooled synthesis data synthesis

RevMan V.5.3.5 will be adapted to synthesize and analyze the eligible data. When *I*^2^ < 50%, a fix-effect model will be used for data synthesis. On the other hand, the random-effects model will be selected to analyze the pooled effect estimates. If a meta-analysis is not possible, we will provide a narrative summary of the results.

#### 2.3.9 Subgroup analysis

If we identify substantial studies, subgroup analysis will be conducted to interpret the heterogeneity across studies, the criteria of a subgroup analysis will be as follows:

1. The age and sex of patients, geographical area
2. Different forms of intervention (different forms of EAHM, frequency, duration, and treatment session).
3. Degree of AV severity.

#### 2.3.10 Sensitivity analysis

After removing low-quality articles, a sensitivity analysis will be conducted to investigate the robustness of the pooled effects, assessing the impact of sample size, heterogeneity quality, statistical model (random-effects or fixed-effects model), and missing data.

#### 2.3.11 Grading the quality of evidence

For grading the strength of evidence from the results obtained, we will use the Grades of Recommendation, Assessment, Development, and Evaluation^[34]^ approach. The evidence level will be classified into 4 possible ratings: very low, low, moderate, or high 4 levels.

#### 2.3.12 Dissemination and ethics

On account of the systematic review based on published evidence, formal ethical approval is not required. It is not necessary to acquire informed consent without patients involved in the study. We are willing to share the results of this review for peer-reviewed journals and present our findings at conferences or a relevant meeting.

## 3 Discussion

The pathogenesis of AV is complex and keeps evolving. The clinical features of AV contain seborrhea, closed or open comedones, pustule, and papule, which contributes to poor appearance and have a severe impact on the patients’ life. The problem of AV has caused wide public concerns over the recent years, the proportion of severity is increasing annually. It not only jeopardizes the body but also has a profound effect on psychological health, such as self-confidence and increased social isolation, even leads to anxiety, depression, and suicide. We should place more emphasis on the AV and take the interdisciplinary approach between psychology and dermatology into account, seeking optimal AV treatment. Nonetheless, scientists have not found satisfying approaches to cure AV so far.

Herbal therapies are our precious wisdom, have been used to cure AV for ages, most herbal products appear to be well tolerated and practical compared with BP. Researches demonstrated that herbal agents, such as topical and oral ayurvedic compounds, oral barberry extract, and gluconolactone solution have significant efficacy in the treatment of AV.^[35–37]^

Herbal medicines originate in China, has been widely applied in clinic for years. The part pathogeny of AV is the massive secretion of sebum and inflammatory. Some studies in China have shown that the EAHM can reduce the secretion of sebum and inhibit inflammation, such as ccutellaria and cortex phellodendri have good inhibition effects on P acnes. The strength of external application of herbal medicine is more economical and safer than CM.^[30,38]^

A review published by Korean Medicine has examined the potential benefits of EAHM in clinical trials. This systematic review and meta-analysis will include many RCTs that were omitted in china databases and some novel well-designed, high-quality, and multi-center RCTs carried out in the last 2 years. The review will provide high-quality evidence-based medicine to determine whether EAMH is an effective and safe intervention for patients with AV.

Nevertheless, we do our utmost to minimize the errors and deviations, despite several restrictions that exist in our systematic review. First, the use of language including English and Chinese, the papers written in other languages such as Japanese may be slipped, which may lead to bias in this review. Second, gender and age of patients, different ingredients of herbal medicine, control interventions, course of treatment, degree of AV severity, and study quality may contribute to heterogeneity arise. Third, it is hard to perform single- or double-blind experiment measures when performing EAHM.

## Author contributions

**Data curation:** Haimin Chen, Zirong Qi, Shujun Shao.

**Supervision:** Yinshan Tang.

**Writing – original draft:** Jin Zhou, Chenglong Jiang.

**Writing – review & editing:** Jin Zhou, Xiaoxiao Li.

Jin Zhou and Chenglong Jiang contributed to the conception of the research. Jin Zhou and Xiaoxiao Li designed the search strategy and drafted the manuscript ultimately. Jin Zhou and Xiaoxiao Li will search all relevant research then select eligible studies independently. Haimin Chen and Zirong Qi will evaluate the bias risk. Shujun Shao is responsible for managing missing data. Yinshan Tang is the arbitrator of this study, in charge of any disagreement and ensure that the review progress smoothly. All the authors who participated in this protocol carefully revised the final manuscript before submission and confirmed the publication of it.
